# Polyethylene wear and cup migration of cemented total hip arthroplasty with femoral heads made of oxidized zirconium, steel, or cobalt chromium: a 10-year secondary analysis from a randomized trial using radiostereometry

**DOI:** 10.2340/17453674.2024.41945

**Published:** 2024-09-30

**Authors:** Håkon Greve JOHANNESSEN, Geir HALLAN, Thomas KADAR, Anne Marie FENSTAD, Stein Håkon Låstad LYGRE, Kristin HAUGAN, Paul Johan HØL, Mona BADAWY, Benedikt JONSSON, Kari INDREKVAM, Arild AAMODT, Ove FURNES

**Affiliations:** 1 Department of Clinical Medicine, Faculty of Medicine, University of Bergen, Bergen, Norway; 2 Department of Physical Medicine and Rehabilitation, Haukeland University Hospital, Bergen, Norway; 3 Norwegian Arthroplasty Register, Department of Orthopaedic Surgery, Haukeland University Hospital, Bergen, Norway; 4 Coastal Hospital in Hagavik, Department of Orthopaedic Surgery, Haukeland University Hospital, Bergen, Norway; 5 Department of Orthopaedic Surgery, St. Olavs Hospital, Trondheim, Norway; 6 Department of Orthopaedic Surgery, Biomatlab, Haukeland University Hospital, Bergen, Norway; 7 Landshospitali University Hospital, Reykjavik, Iceland; 8 Department of Orthopaedic Surgery, Lovisenberg Diakonal Hospital, Oslo, Norway; 9 Department of Occupational Medicine, Haukeland University Hospital, Bergen, Norway

## Abstract

**Background and purpose:**

We aimed to evaluate polyethylene (PE) wear, cup migration, and clinical outcome over 10 years in total hip arthroplasties (THA) using different articulations.

**Methods:**

This is a secondary analysis of 150 patients randomized into 5 groups, using different articulations: Charnley/Charnley Ogee for steel and conventional polyethylene (CPE), or Spectron EF/Reflection with either CPE or highly cross linked polyethylene (XLPE) cups, paired with heads made of either cobalt-chromium (CoCr) or oxidized zirconium (OxZr). All cups were cemented. Patients underwent repeated radiostereometric analysis (RSA) measurements for up to 10 years to assess wear and migration. Clinical outcome was assessed using Harris Hip Score (HHS).

**Results:**

After 10 years, the XLPE cups demonstrated low wear rates: 0.08 mm (95% confidence interval [CI] –0.11 to 0.26 mm) with CoCr heads and 0.06 mm (CI –0.14 to 0.26 mm) with OxZr heads, with a mean difference of 0.01 mm (CI –0.26 to 0.29 mm). In contrast, CPE cups exhibited significantly more wear: 1.35 mm (CI 1.16 to 1.55 mm) with CoCr heads and 1.68 mm (CI 1.44 to 1.92 mm) with OxZr heads, with a mean difference of 0.33 mm (CI 0.02 to 0.64 mm). The Charnley/Ogee group (CPE) showed PE wear of 0.34 mm (CI 0.12 to 0.56 mm). The CPE groups with OxZr and CoCr heads had 0.67 mm (CI 0.38 to 0.96 mm) and 0.35 mm (CI 0.09 to 0.61 mm) greater proximal migration respectively than the corresponding XLPE groups. HHS was similar across all groups.

**Conclusion:**

We found no significant advantage of OxZr over CoCr heads in reducing wear or migration. XLPE demonstrated a major reduction in wear as well as a reduction in cup migration compared with CPE. Charnley performed better than the other CPE cups in terms of PE wear and cup migration. No differences in clinical outcome were found.

Aseptic loosening is a major reason for failure of total hip replacements, and one of the main contributors to this is osteolysis induced by wear particles from the polyethylene (PE) articulating surface [1]. Polyethylene with a substantial degree of crosslinking (XLPE) has displayed notable improvements in wear resistance when compared with conventional polyethylene (CPE) [2-5].

Oxidized zirconium (OxZr), or Oxinium (Smith+Nephew, Memphis, TN, USA) is a femoral head material created by heating a zirconium–niobium alloy, forming a 5-micron thick ceramic zirconium-oxide surface. It aims for metal strength and ceramic wear resistance. There is no long term clinical evidence of enhanced wear characteristics [6].

The 2-year wear and migration data from the present study has been published and this is a secondary analysis with 10 years’ follow-up [7] using RSA, which is requested [8].

The primary aim of our study was to compare the effect of different PE types and different femoral head materials on PE wear in cemented THA at 10 years. Secondary aims were to compare cup migration and clinical outcomes.

## Methods

Between November 2004 and June 2007, 150 patients (70% female), with a mean age of 70 years (range 59–80), were recruited to total hip arthroplasty (THA), addressing primary or secondary hip osteoarthritis. All patients signed an informed consent form before inclusion. In bilateral cases, only 1 hip was included. Exclusion criteria were body mass index (BMI) > 35, uncompensated cardio-pulmonary disease, malignant disease, dementia, rheumatoid arthritis, or any other serious systemic diseases.

Preoperative pelvic radiographs, assessed with templates, determined inclusion if both implant systems in the study could restore hip biomechanics adequately.

The patients were randomly assigned into 1 of 5 groups. 8 consultant orthopedic surgeons and 1 resident orthopedic surgeon were responsible for the patient selection and surgery. To prevent surgeon-related bias and ensure equitable group distribution, block randomization at the surgeon level was used. This method involved sealed envelopes containing the assigned intervention group and maintained the blinding of patients to their treatment group throughout the study.

### Intervention

The following PEs were used: conventional polyethylene (CPE) in the form of ultra-high molecular weight polyethylene (UHMWPE) and XLPE.

The 5 groups consisted of the following cemented THAs:

1. Steel/CPE: Charnley monoblock stainless steel femoral stem with a 22.2 mm head and a Charnley Ogee UHMWPE (GUR 1050) acetabular cup that was γ-sterilized with 2.5 Mrad in nitrogen.
2. CoCr/CPE: Spectron EF femoral stem with a 28 mm CoCr femoral head and a Reflection All-Poly UHMWPE (GUR 1050) cup that was not irradiated but sterilized by ethylene oxide (EtO).
3. OxZr/CPE: Spectron EF femoral stem with a 28 mm Oxinium femoral head and a Reflection All-Poly UHMWPE (GUR 1050) cup that was not irradiated but EtO sterilized.
4. CoCr/XLPE: Spectron EF femoral stem with a 28 mm CoCr femoral head and a Reflection All-Poly XLPE (GUR 1050) cup irradiated with 10 Mrad, melted at 135°C, and EtO sterilized.
5. OxZr/XLPE: Spectron EF femoral stem with a 28 mm Oxinium femoral head and a Reflection All-Poly XLPE (GUR 1050) cup irradiated with 10 Mrad, melted at 135°C, and EtO sterilized.

The acetabular cups were supplied with tantalum markers embedded in the dome and edge by the manufacturer (Reflection, Smith+Nephew and Charnley, DePuy Synthes, Warsaw, IN, USA). The Charnley cups had 10 x 0.8-mm markers and the Reflection cups had 6 x 1.0-mm markers. During surgery 6–9 markers were inserted in the periprosthetic pelvic bone. The Charnley group had 1 mm markers inserted in the periprosthetic bone, and the Reflection/Spectron-EF groups had 0.8 mm markers.

The surgical technique and postoperative treatment were standardized and have been described in detail in a previous paper [7].

### Outcome measures

The median time for the first postoperative RSA examination was 11 (9–15) days after surgery, and further examinations were conducted at 3, 6, 12, 60, and 120 months after surgery. All examinations were performed by the same radiographer. The RSA technique was standardized according to guidelines and has been described in detail previously [7,9]. The movement of the center of the femoral head, represented as a point, was used to determine the cup penetration, using the tantalum markers in the PE as a fixed reference. Similarly, the movement of the rigid body made by the markers in the PE with the periprosthetic bone as reference represented cup migration. Penetration, and cup translation and rotation were calculated along and around the horizontal (X), longitudinal (Y), and sagittal axes (Z) based on signed values and were computed using the UmRSA Digital measure version 5.0 software (RSA Biomedical, Umeå, Sweden). Our main outcome was wear of the acetabular PE cup, defined as the proximal head penetration (proximal translation of the femoral head along the Y-axis) from the 1-year follow-up to the 10-year follow-up [10]. We also report the total head penetration (from postoperative to 10-year follow-up) and the annual wear rate (rate of wear per year from 1-year to 10-year follow-up). Cup translation along and rotation around the 3 axes at 10 years are presented as secondary outcomes with proximal translation along the Y-axis considered to be the most clinically relevant migration outcome.

RSA measurements required 3 or more identifiable markers in the cup for penetration and in both the cup and periprosthetic bone for migration measurements. The upper limit for the mean error of body-fitting (ME) was set to 0.35 and the condition number (CN) at 150 [9].

The precision of the RSA measurements was done by calculating the difference between double RSA examinations for 50 patients at 1-year follow-up and gave a precision level of 0.1–0.2 mm for cup translation, 0.4–0.5° for cup rotation, and 0.1 mm for femoral head penetration. The calculations were described in detail in a previous paper [7].

Harris Hip Score (HHS) was a secondary outcome used to assess clinical outcomes comparing preoperative scores with postoperative scores at the same intervals as the RSA examinations [11]. Scores were conducted by a surgeon or physiotherapist.

Results from the RSA measurements as well as the clinical outcome were analyzed as intention to treat.

### Statistics

After a power analysis we opted for a group size of 30 individuals [7,12]. Demographic differences were evaluated using chi-square tests and one-way ANOVA. Due to the repeated measurement design with some incomplete outcome data, we used linear mixed-effects models when analyzing PE wear and cup migration. 2 time variables were used, 1 starting postoperatively, and the other starting at the 1-year follow-up to account for bedding in. Annual wear rates from 1 to 10 years were calculated by dividing the amount of wear between the 1-year and the subsequent follow-ups by the amount of time between these follow-ups, and then analyzed with the linear mixed model. A pairwise comparison was done for PE wear from 1–10 years and proximal Y-migration from postoperatively to 10-year follow-up. We did not adjust for multiple comparisons. Clinical outcomes with HHS were compared between the groups using the Kruskal–Wallis test. Changes in HHS from preoperatively to 10 years were analyzed with the Wilcoxon signed rank test. All tests were 2-sided, with significance level set at 0.05. Statistics were compiled using SPSS version 26 (IBM Corp, Armonk, NY, USA), and STATA version 18 (StataCorp, College Station, TX, USA) for the linear mixed-effects models. The R 4.2.2 statistical software package (R Foundation for Statistical Computing, Vienna, Austria) was used for creating the figures.

The CONSORT reporting guideline on multi-arm parallel-group randomized trials was followed [13]. In addition, the new RSA guideline was consulted [14].

### Ethics, registration, data sharing plan, use of AI, funding, and disclosures

The study was registered in ClinicalTrials.Gov (NCT00698672) and approved by the Western Norway regional ethics committee (REK number 2014-02370). Deidentified data might be shared upon request. ChatGPT (OpenAI, San Francisco, CA, USA) was used in attempt to shorten sections of the abstract. The study was jointly financed by OrthoMedic AS, Smith & Nephew Norway AS, and the Regional Health Board of Western Norway. The first author received a grant from the University of Bergen for being part of the medical student research program. None of the funding sources played any role in the preparation, performance, or analysis of the results of this study. GH has received speaker fees from Ortomedic and LINK Norway. OF has received fees for lectures on cementing technique for knee replacement given to Heraeus Medical and Ortomedic. PJH has also received fees for lectures from Heraeus Medical. Complete disclosure of interest forms according to ICMJE are available on the article page, doi: 10.2340/17453674.2024.41945

## Results

**Figure 1:**
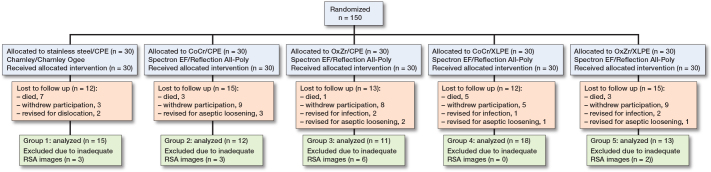
Flowchart of patients with cemented total hip arthroplasty with 5 different articulations, the number of patients included, and the number of patients remaining for RSA analysis at 10 years, with reasons for exclusion at the 10-year follow-up noted. For detailed description of study groups, see text. CPE = conventional polyethylene; CoCr = cobalt-chrome; OxZr = oxidized zirconium; XLPE = highly crosslinked polyethylene.

During the 10-year study period, 19 patients died, and 34 patients did not attend the 10-year follow-up due to health issues or unwillingness to participate further. Furthermore, 2 patient were excluded from the 10-year RSA evaluation due to dislocation, 5 patients for infection, and 7 underwent revisions due to aseptic loosening before the 10-year follow-up (Figure 1). Another 14 patients were excluded from cup-wear assessments due to technical issues associated with the images, such as loss of tantalum markers, high ME, or high CN (Figure 1). An additional 2 patients, 1 each from the CPE/OxZr and XLPE/OxZr groups, were excluded from cup-migration analysis, for similar technical reasons. There were no differences in demographic characteristics between the groups at baseline other than cup size, due to the Charnley Ogee cup being used only in smaller sizes, i.e. 40 and 43 mm (Table 1).

**Table 1:** Cemented total hip arthroplasty with 5 different articulations: demographics of the patients at baseline. Values are mean (standard deviation) unless otherwise specified

Factor	Steel/CPE ^a^	CoCr/CPE ^b^	OxZr/CPE ^b^	CoCr/XLPE ^b^	OxZr/XLPE ^b^
	n = 30	n = 30	n = 30	n = 30	n = 30
Female/male, n	20/10	20/10	23/7	20/10	22/8
Age	70 (6.1)	69 (5.9)	69 (6.7)	70 (5.3)	70 (5.4)
Weight, kg	76 (15.0)	76 (11.1)	72 (13.9)	80 (14.8)	76 (14.6)
BMI	26.4 (3.9)	26.0 (2.2)	25.9 (3.3)	27.6 (4.1)	26.7 (4.0)
Primary/secondary OA, n	28/2	26/4	26/4	22/8	27/3
Preoperative HHS	45	42	47	47	40
Median cup size, mm (range)	43 (40–43)	52 (49–61)	52 (49–58)	52 (46–58)	52 (43–58)

For abbreviations, see Legend to Figure 1.

a Charnley/Ogee.

b Spectron EF/Reflection.

### Wear

**Figure 2:**
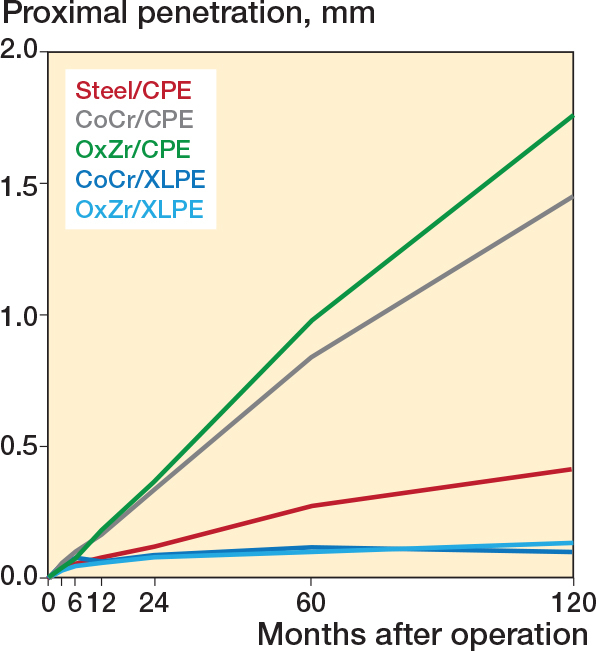
Cemented total hip arthroplasty with 5 different articulations and the proximal penetration of their femoral heads into the acetabular cup (mm) along the Y-axis over 10 years. For abbreviations, see Legend to Figure 1.

For the primary outcome of PE wear from 1–10 years, the choice of femoral head material, OxZr over CoCr, gave no discernible reduction in PE wear for the 2 Reflection cups (CPE and XLPE) (Table 2, Figure 2). Over this period the XLPE cups had PE wear of 0.08 mm (CI –0.11 to 0.26 mm) with CoCr heads and 0.06 mm (CI –0.14 to 0.26 mm) with OxZr heads, while the CPE cups had 1.35 mm (CI 1.16 to 1.55 mm) and 1.68 mm (CI 1.44 to 1.92 mm) with CoCr and OxZr respectively. The Charnley/Ogee had PE wear of 0.34 mm (CI 0.12 to 0.56 mm), slightly higher than the XLPE cups, but lower than the Reflection CPE cups (Table 3). Over the course of 1 to 10 years, the XLPE cups had a difference in PE wear of 0.01 mm (CI –0.26 to 0.29 mm) with the 2 head materials. With CPE cups there was a 0.33 mm (CI 0.02 to 0.64 mm) increase in PE wear with OxZr over CoCr heads (Table 3).

**Table 2:** Cemented total hip arthroplasty with 5 different articulations showing total head penetration from postoperatively to 2-, 5-, and 10-year follow-up for each study group. Also includes “bedding-in,” annual wear rate, and head penetration from 1-year to 10-year follow-up. Values are mean with 95% confidence intervals, the results from the linear mixed model

Factor	Steel/CPE ^a^	CoCr/CPE ^b^	OxZr/CPE ^b^	CoCr/XLPE ^b^	OxZr/XLPE ^b^
Total head penetration, mm ^c^					
2 years	0.13 (0.03–0.23)	0.34 (0.27–0.42)	0.36 (0.28–0.44)	0.09 (0.02 to 0.16)	0.08 (–0.00 to 0.16)
5 years	0.26 (0.13–0.39)	0.85 (0.75–0.95)	1.00 (0.89–1.11)	0.12 (0.02 to 0.22)	0.10 (–0.02 to 0.21)
10 years	0.42 (0.25–0.58)	1.47 (1.33–1.61)	1.81 (1.66–1.96)	0.12 (–0.01 to 0.25)	0.13 (–0.02 to 0.27)
Bedding-in, mm ^d^	0.09 (0.05–0.13)	0.16 (0.13–0.20)	0.19 (0.15–0.23)	0.06 (0.03 to 0.09)	0.06 (0.02 to 0.09)
Total wear from 1 year, mm ^e^	0.34 (0.12–0.56)	1.35 (1.16–1.55)	1.68 (1.44–1.92)	0.08 (–0.11 to 0.26)	0.06 (–0.14 to 0.26)
Annual wear rate in mm ^f^	0.04 (0.01–0.06)	0.15 (0.12–0.17)	0.18 (0.15–0.21)	0.007 (–0.015 to 0.03)	0.007 (–0.017 to 0.03)

For abbreviations, see Figure 1.

a Charnley/Ogee.

b Spectron EF/Reflection

c Total head penetration from postoperatively until the given year.

d Head penetration from postoperatively to 1-year follow-up.

e Wear from 1- to 10-year follow-up, thus excluding bedding-in.

f Wear rate, calculated as annual wear rate from 1-year to 10-year follow-up.

**Table 3:** Cemented total hip arthroplasty with 5 different articulations: pairwise comparison of differences in 1-to-10-year mean PE wear in mm with 95% confidence interval results from the linear mixed model

1-to-10-year mean PE wear, mm				Difference
Reference		Comparison ^b^		comparison–reference
Steel/CPE ^a^	0.34 (0.12 to 0.56)	CoCr/CPE	1.35 (1.16 to 1.55)	1.01 (0.71 to 1.31)
Steel/CPE ^a^	0.34 (0.12 to 0.56)	OxZr/CPE	1.68 (1.44 to 1.92)	1.34 (1.01 to 1.67)
Steel/CPE ^a^	0.34 (0.12 to 0.56)	CoCr/XLPE	0.08 (–0.11 to 0.26)	–0.27 (–0.55 to 0.02)
Steel/CPE ^a^	0.34 (0.12 to 0.56)	OxZr/XLPE	0.06 (–0.14 to 0.26)	–0.28 (–0.58 to 0.02)
CoCr/CPE ^b^	1.35 (1.16 to 1.55)	OxZr/CPE	1.68 (1.44 to 1.92)	0.33 (0.02 to 0.64)
CoCr/CPE ^b^	1.35 (1.16 to 1.55)	CoCr/XLPE	0.08 (–0.11 to 0.26)	–1.28 (–1.54 to –1.01)
CoCr/CPE ^b^	1.35 (1.16 to 1.55)	OxZr/XLPE	0.06 (–0.14 to 0.26)	–1.29 (–1.57 to –1.01)
OxZr/CPE ^b^	1.68 (1.44 to 1.92)	CoCr/XLPE	0.08 (–0.11 to 0.26)	–1.60 (–1.90 to –1.30)
OxZr/CPE ^b^	1.68 (1.44 to 1.92)	OxZr/XLPE	0.06 (–0.14 to 0.26)	–1.62 (–1.93 to –1.30)
CoCr/XLPE ^b^	0.08 (–0.11 to 0.26)	OxZr/XLPE	0.06 (–0.14 to 0.26)	–0.01 (–0.29 to 0.26)

For abbreviations, see Legend to Figure 1.

a Charnley/Ogee.

b Spectron EF/Reflection.

### Migration

**Figure 3:**
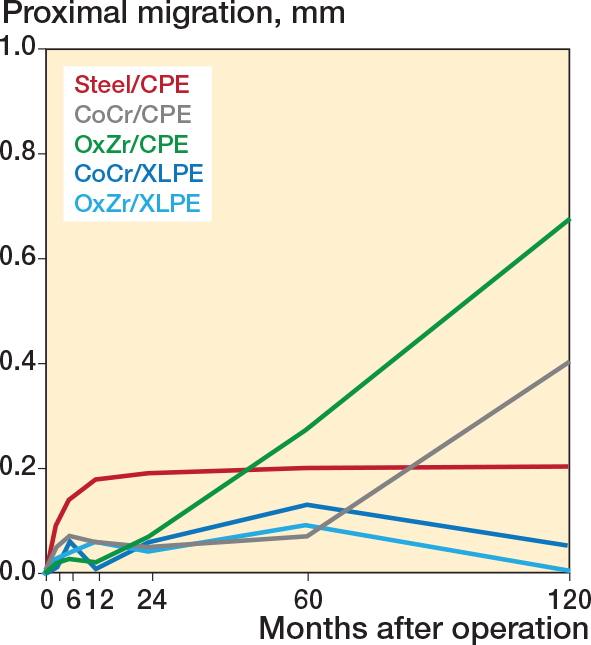
Cemented total hip arthroplasty with 5 different articulations and the proximal migration of their acetabular cups (mm) along the Y-axis over 10 years for the 5 study groups.

The Charnley cups had more proximal Y-translation in the first year compared with the Reflection cups, but from 1 year until 10 years they remained stable (Figure 3 and Table 4, see Appendix). The XLPE cups with CoCr or OxZr heads were stable from 3 months until the 10-year measurements and there was no difference between the 2 articulations (Table 5 and Figure 3). The 2 groups with Reflection CPE migrated more than the 2 XLPE groups at 10 years. The OxZr group tended to migrate more than the CoCr group (Tables 4–5 and Figure 3).

**Table 4:** Cemented total hip arthroplasty with 5 different articulations: mean cup migration in mm and degrees for all study groups across the study period with 95% confidence interval. Point estimates for the different follow-up timepoints created with the linear mixed model. Values in brachets are cases available at each time point

Follow-up, months	Steel/CPE ^a^	CoCr/CPE ^b^	OxZr/CPE ^b^	CoCr/XLPE ^b^	OxZr/XLPE ^b^
X-translation					
3	0.00 (–0.08 to 0.08) [23]	0.04 (–0.04 to 0.12) [20]	0.05 (–0.05 to 0.14) [15]	0.00 (–0.07 to 0.08) [26]	0.02 (–0.06 to 0.10) [21]
6	–0.01 (–0.09 to 0.06) [25]	0.02 (–0.06 to 0.10) [22]	0.03 (–0.07 to 0.13) [14]	–0.03 (–0.11 to 0.05) [24]	0.03 (–0.05 to 0.12) [20]
12	0.01 (–0.07 to 0.09) [27]	0.07 (–0.02 to 0.16) [21]	0.07 (–0.03 to 0.17) [16]	0.03 (–0.05 to 0.11) [27]	0.03 (–0.06 to 0.11) [24]
24	–0.05 (–0.14 to 0.04) [27]	0.09 (–0.00 to 0.19) [24]	0.08 (–0.02 to 0.18) [21]	0.01 (–0.08 to 0.10) [29]	0.03 (–0.06 to 0.13) [24]
60	–0.02 (–0.12 to 0.09) [25]	0.17 (0.06 to 0.28) [24]	0.00 (–0.12 to 0.13) [19]	–0.11 (–0.22 to 0.00) [22]	–0.04 (–0.17 to 0.08) [17]
120	0.01 (–0.14 to 0.15) [15]	0.09 (–0.06 to 0.25) [12]	–0.30 (–0.47 to –0.13) [10]	–0.02 (–0.15 to 0.12) [18]	0.08 (–0.08 to 0.24) [12]
Y-translation					
3	0.09 (0.01 to 0.17)	0.05 (–0.04 to 0.13)	0.02 (–0.08 to 0.12)	0.01 (–0.06 to 0.09)	0.03 (–0.05 to 0.11)
6	0.14 (0.07 to 0.22)	0.07 (–0.01 to 0.16)	0.03 (–0.07 to 0.13)	0.06 (–0.02 to 0.14)	0.04 (–0.05 to 0.13)
12	0.18 (0.09 to 0.26)	0.06 (–0.04 to 0.16)	0.02 (–0.09 to 0.13)	0.01 (–0.07 to 0.10)	0.06 (–0.04 to 0.15)
24	0.19 (0.08 to 0.30)	0.05 (–0.06 to 0.17)	0.07 (–0.05 to 0.19)	0.06 (–0.04 to 0.17)	0.04 (–0.07 to 0.16)
60	0.20 (0.06 to 0.33)	0.07 (–0.07 to 0.21)	0.27 (0.11 to 0.42)	0.13 (–0.01 to 0.27)	0.09 (–0.06 to 0.25)
120	0.20 (0.02 to 0.38)	0.40 (0.20 to 0.59)	0.67 (0.46 to 0.88)	0.05 (–0.13 to 0.22)	–0.00 (–0.20 to 0.20)
Z-translation					
3	0.06 (–0.02 to 0.13)	–0.00 (–0.08 to 0.08)	0.07 (–0.03 to 0.16)	0.02 (–0.05 to 0.10)	0.01 (–0.06 to 0.09)
6	0.03 (–0.04 to 0.11)	0.04 (–0.05 to 0.12)	0.04 (–0.06 to 0.14)	0.00 (–0.08 to 0.08)	0.03 (–0.06 to 0.11)
12	0.04 (–0.04 to 0.13)	0.03 (–0.07 to 0.12)	0.08 (–0.02 to 0.19)	–0.01 (–0.10 to 0.07)	0.00 (–0.09 to 0.09)
24	0.03 (–0.07 to 0.12)	0.05 (–0.05 to 0.15)	0.11 (0.00 to 0.22)	0.01 (–0.09 to 0.10)	0.05 (–0.05 to 0.16)
60	–0.09 (–0.20 to 0.02)	0.07 (–0.05 to 0.18)	0.07 (–0.06 to 0.19)	0.05 (–0.06 to 0.17)	0.01 (–0.12 to 0.14)
120	–0.08 (–0.22 to 0.06)	0.01 (–0.14 to 0.17)	0.08 (–0.08 to 0.25)	–0.05 (–0.18 to 0.09)	0.14 (–0.01 to 0.30)
X-rotation					
3	–0.13 (–0.28 to 0.02)	0.01 (–0.15 to 0.18)	–0.04 (–0.22 to 0.15)	–0.12 (–0.27 to 0.02)	–0.06 (–0.21 to 0.10)
6	–0.08 (–0.24 to 0.07)	–0.09 (–0.26 to 0.08)	–0.09 (–0.28 to 0.11)	–0.01 (–0.17 to 0.15)	–0.11 (–0.28 to 0.06)
12	–0.22 (–0.39 to –0.04)	–0.07 (–0.26 to 0.12)	–0.20 (–0.41 to 0.01)	–0.12 (–0.30 to 0.05)	0.04 (–0.14 to 0.23)
24	–0.14 (–0.34 to 0.06)	–0.08 (–0.29 to 0.14)	–0.33 (–0.55 to –0.10)	–0.07 (–0.27 to 0.13)	0.01 (–0.21 to 0.22)
60	–0.16 (–0.40 to 0.08)	–0.09 (–0.34 to 0.15)	–0.37 (–0.63 to –0.10)	–0.31 (–0.56 to –0.07)	0.03 (–0.24 to 0.30)
120	–0.03 (–0.33 to 0.28)	–0.30 (–0.62 to 0.03)	–0.09 (–0.44 to 0.26)	0.09 (–0.20 to 0.38)	–0.23 (–0.56 to 0.10)
Y-rotation					
3	0.12 (–0.10 to 0.35)	0.12 (–0.11 to 0.35)	–0.01 (–0.28 to 0.26)	–0.04 (–0.24 to 0.17)	0.04 (–0.18 to 0.26)
6	0.11 (–0.09 to 0.32)	0.10 (–0.12 to 0.32)	0.12 (–0.15 to 0.39)	0.14 (–0.07 to 0.35)	0.03 (–0.19 to 0.26)
12	0.09 (–0.13 to 0.30)	0.20 (–0.03 to 0.44)	0.15 (–0.12 to 0.42)	0.16 (–0.06 to 0.37)	0.14 (–0.09 to 0.37)
24	0.03 (–0.21 to 0.27)	0.10 (–0.16 to 0.35)	0.27 (–0.01 to 0.54)	0.15 (–0.09 to 0.38)	0.06 (–0.20 to 0.32)
60	0.11 (–0.18 to 0.40)	0.14 (–0.16 to 0.43)	0.19 (–0.13 to 0.52)	0.08 (–0.22 to 0.37)	0.08 (–0.25 to 0.41)
120	0.14 (–0.25 to 0.52)	0.16 (–0.26 to 0.58)	0.89 ( 0.43 to 1.34)	0.24 (–0.12 to 0.60)	–0.07 (–0.50 to 0.36)
Z-rotation					
3	0.02 (–0.20 to 0.25)	0.10 (–0.14 to 0.34)	0.22 (–0.05 to 0.50)	0.04 (–0.17 to 0.26)	0.11 (–0.12 to 0.34)
6	0.03 (–0.18 to 0.24)	0.11 (–0.11 to 0.33)	0.15 (–0.12 to 0.42)	0.03 (–0.19 to 0.24)	0.13 (–0.10 to 0.36)
12	0.18 (–0.04 to 0.41)	0.26 (0.01 to 0.51)	0.21 (–0.07 to 0.48)	0.11 (–0.11 to 0.33)	0.05 (–0.18 to 0.29)
24	0.08 (–0.18 to 0.34)	0.26 (–0.02 to 0.54)	0.28 (–0.01 to 0.58)	0.18 (–0.08 to 0.45)	0.10 (–0.18 to 0.38)
60	0.29 (–0.04 to 0.62)	0.42 ( 0.09 to 0.76)	0.53 (0.16 to 0.90)	0.18 (–0.16 to 0.52)	0.01 (–0.36 to 0.39)
120	0.35 (–0.09 to 0.79)	0.36 (–0.12 to 0.83)	1.99 (1.48 to 2.51)	0.24 (–0.18 to 0.66)	0.11 (–0.37 to 0.60)

For abbreviations, see Legend to Figure 1.

a Charnley/Ogee.

b Spectron EF/Reflection.

**Table 5:** Cemented total hip arthroplasty with 5 different articulations: pairwise comparison of differences in proximal migration along the Y-axis with results from the linear mixed model

Mean proximal migration from postoperative to 10 years, mm				Difference
Reference		Comparison ^b^		comparison–reference
Steel/CPE ^a^	0.20 (0.02 to 0.38)	CoCr/CPE	0.40 (0.2 to 0.59)	0.20 (–0.07 to 0.46)
Steel/CPE ^a^	0.20 (0.02 to 0.38)	OxZr/CPE	0.67 (0.46 to 0.88)	0.47 (0.19 to 0.74)
Steel/CPE ^a^	0.20 (0.02 to 0.38)	CoCr/XLPE	0.05 (–0.13 to 0.22)	–0.15 (–0.40 to 0.09)
Steel/CPE ^a^	0.20 (0.02 to 0.38)	OxZr/XLPE	–0.002 (–0.2 to 0.2)	–0.20 (–0.47 to 0.07)
CoCr/CPE ^b^	0.40 (0.20 to 0.59)	OxZr/CPE	0.67 (0.46 to 0.88)	0.27 (–0.02 to 0.56)
CoCr/CPE ^b^	0.40 (0.20 to 0.59)	CoCr/XLPE	0.05 (–0.13 to 0.22)	–0.35 (–0.61 to –0.09)
CoCr/CPE ^b^	0.40 (0.20 to 0.59)	OxZr/XLPE	–0.002 (–0.2 to 0.2)	–0.40 (–0.68 to –0.12)
OxZr/CPE ^b^	0.67 (0.46 to 0.88)	CoCr/XLPE	0.05 (–0.13 to 0.22)	–0.62 (–0.96 to –0.35)
OxZr/CPE ^b^	0.67 (0.46 to 0.88)	OxZr/XLPE	–0.002 (–0.2 to 0.2)	–0.67 (–0.96 to –0.38)
CoCr/XLPE ^b^	0.05 (–0.13 to 0.22)	OxZr/XLPE	–0.002 (–0.2 to 0.2)	–0.05 (–0.31 to 0.22)

For abbreviations, see Legend to Figure 1.

a Charnley/Ogee.

b Spectron EF/Reflection.

### Clinical outcome

Patients at the 10-year follow-up showed significant HHS improvement across all groups, increasing from a preoperative 44 (SD 15) to 91 (SD 10) at 2 years to 87 (SD 15) at 10 years, with no significant differences between the groups.

## Discussion

We aimed to evaluate wear and migration over 10 years in total hip arthroplasties (THA) using different articulations.

We found for our primary outcome high levels of wear for the Reflection CPE cups contrary to low levels of wear of the XLPE cups, which was below the 0.1 mm detection limit of the RSA measurements between 1 and 10 years. Notably, OxZr heads did not protect against PE wear compared with CoCr heads. For our secondary outcome we found that the Reflection CPE cups migrated more than the XLPE and the Charnley cups between 5 years to 10 years.

### Wear

A wear rate > 0.1 mm/year, equivalent to more than 1 mm of total wear over a 10-year period, has been identified as a factor contributing to an elevated risk of osteolysis [15]. We found that the study groups with the CPE Reflection All-Poly cup had a wear rate above this threshold. This particular cup has demonstrated poor outcomes in several previous studies [7,12,16,17].

In the XLPE groups, the average 10-year wear remained below our study’s detection limit for proximal head penetration (0.1 mm). This reduced wear associated with XLPE has also been shown in a scoping review with similar lengths of follow-up [10].

The Charnley/Ogee cup (CPE) showed slightly higher wear than the XLPE cups. Given the smaller head diameter of the Charnley prosthesis and viewing wear as a volumetric parameter, and a wear rate below 0.1 mm/year, this disparity may not be of significant concern. The slight levels of radiation (2.5 Mrad) used for the sterilization of the Charnley cup have probably given some level of crosslinking to the PE and could help explain the longevity of this prothesis and the superior performance over the Reflection All-Poly CPE cup that had no crosslinking. These findings align with the results obtained from the same study at 2-year follow-up [7,12].

Additionally, there was no discernible reduction in wear associated with OxZr heads compared with the CoCr heads, when they articulated with XLPE. This observation is consistent with the findings from another clinical RSA 10-year follow-up study [18]. Furthermore, this aligns with the results obtained from the 5-year follow-up within the present study [12]. However, we found a possible increase in PE wear with OxZr over CoCr when articulating with CPE, which was not present at earlier follow-up. This is probably of minor clinical significance, considering XLPE has taken over as the preferable PE in THA, and there not being a difference with XLPE. This indicates that the choice of PE had a higher impact on the PE-wear than the head material. This could, however, be a type 1 error, as the analysis did not include adjustments for multiple comparisons and the number of hips with 10 years’ measurements was low especially in the OxZr group with CPE. Registry studies have shown that THA with OxZr heads combined with highly crosslinked PE has demonstrated favorable comparative performance in terms of risk of revision [19,20]. However, these studies included a variety of stem and cup designs, making it challenging to ascertain whether the observed results were primarily caused by the femoral head material or the specific stem and cup designs.

### Cup migration

The Reflection CPE cups migrated more from 5 to 10 years’ measurement compared with the XLPE and Charnley cups. The OxZr group showed this trend even from the 2 year measurements. Most of the migration values corresponded with the precision of the RSA measurement. At the 2-year follow-up the only difference in migration between the groups was that the Charnley group had higher proximal migration compared with the rest [7]. This was no longer the case at 10 years and the Charnley cups were stable from 1 year to 10 years’ measurements as were the Reflection XLPE cups. The CPE cups had higher PE wear than the XLPE groups and the Charnley group, with increased levels of PE particles contributing to osteolysis and loosening aligning with the dose/response theory [21]. More cups were also revised for loosening in the CPE groups than the XLPE groups. As previously mentioned, it is important to emphasize that our study exclusively examined THAs that remained unrevised at 10 years. Therefore, it is plausible to argue that the difference in migration would have been higher if we had RSA measurements right before revision for the cases that underwent revisions.

### Limitations

A relatively low number of patients remained in the study at the 10-year follow-up, primarily due to factors such as patient mortality, comorbidities, or unwillingness to continue participating, in addition to missing or excluded RSA measurements. Also, the power analysis was done with 2-year follow-up in mind. The median time for the postoperative RSA examination was 11 days. One could argue that this prolonged period of load on the hip could influence the RSA results. However, this period was similar for all 5 groups. The RSA examinations were done in a supine position, and this could give less contact between the head and cup compared with a standing examination, but a study comparing this found no such difference [22]. Patients who underwent revision were excluded from RSA follow-up. Consequently, the THAs that were measured at 10 years were presumably in the patients with the best overall health and outcomes. Nonetheless, it is crucial to highlight that comprehensive patient follow-up, encompassing data related to death and revision surgery, was diligently conducted and tracked until 10 years’ follow-up of all patients. Consulting the 5 domains from the Cochrane risk of bias tool 2, possible performance and detection bias could have happened due to the blinding procedure [23]. The intervention group was known to the surgeons and personnel conducting the follow-up, and some patients probably learned their intervention during follow-up. However, it seems unlikely this affected the outcome measurements, except for perhaps HHS. Attrition bias could be possible due to missing values at certain follow-ups. The reason for missing values is reported and taken into account with the linear mixed model.

As the last patients were included in 2007, RSA follow-up after 10 years could be possible. However, this will not be done in this study. Considering the number of patients lost to follow-up at 10 years, and the patients’ age at inclusion, it is likely that more patients would be lost. RSA studies with longer than 10 years’ follow-up should probably be encouraged considering the increased longevity of new implants and materials, and the potential of migration or wear patterns changing over time, as here, where the Charnley cups had more proximal migration at 2 years, but remained more stable than the Reflection CPE cups at later follow-ups. If done, care should be taken to secure as few dropouts as possible by including younger age groups than we did.

### Conclusion

The present study did not reveal any advantages of OxZr over CoCr heads with respect to reducing PE wear, cup migration, or better clinical outcomes at 10 years’ follow-up. XLPE demonstrated excellent performance, with virtually imperceptible wear over the study period, as well as reducing cup migration compared with CPE. The Charnley prosthesis with steel and CPE had more PE wear than the Reflection XLPE cups, but performed better than the Reflection CPE cups in terms of both PE wear and cup migration. The combination of CoCr and XLPE seems to be an effective, reasonable and safe choice for THA, considering the increased costs of OxXr.  

## Notes

HGJ: data collection, analysis, and writing. GH, TK, OF: planning, data collection, and revision of the manuscript. AMF, AA, and KH: data collection, analysis, and revision of the manuscript. MB, BJ, and KI: data collection and revision of the manuscript. SHLL: data analysis and revision of the manuscript. PJH: revision of the manuscript.

Special thanks are extended to Terje Stokke, who supplied all the radiographs, and all the surgeons who contributed to the study.

Handling co-editors: Bart A Swierstra and Robin Christensen

*Acta* thanks Dennis Janssen and Bart G Pijls for help with peer review of this manuscript.
